# Migration of Medical Doctors from Nepal: Analyzing Trends and Policy Implications

**DOI:** 10.31729/jnma.8750

**Published:** 2024-09-30

**Authors:** Lochan Karki, Bikrant Dhakal, Kailash Kumar Bhandari, Suzit Bhusal, Ashlesha Chaudhary

**Affiliations:** 1 Nepal Medical Association, Siddhisadan, Kathmandu, Nepal; 2 Nepal Medical Council, Bansbari, Kathmandu, Nepal; 3 Nepal Orthopedic Hospital, Gokarneshwor, Kathmandu, Nepal; 4 Research and Development Unit, National Trauma Center, Bir Hospital, Kathmandu, Nepal

**Keywords:** *migration*, *healthcare professionals*, *Nepal*, *trend analysis*

## Abstract

One way migration of health care professionals especially from low-income countries like Nepal has become a concerning issue where the already fragile health system of the country faces challenges of losing skilled health care professionals to high-income countries. This trend analysis provides insights into the dynamics of Nepal's healthcare workforce migration, it offers a tailored understanding of factors influencing migration patterns, and its impact on the genera! population of the country and globally. It helps formulate targeted policies for workforce retention, resource planning, and addressing the country's healthcare needs and comparison with the global context. Analyzing the migration trends of medical professionals enhances medical literature by offering a comprehensive understanding of the factors affecting healthcare systems, public health, economics, education, and policy development. This knowledge plays a vital role in shaping future policies in the field of medicine in Nepal.

## INTRODUCTION

Nepal has an unequal distribution of health workers leading to a shortage of health workers in most parts of the country. From 2011 to 2019, Nepal had a doctor density of 8.1 per 10,000 population, while the density of midwifery and nursing professionals was 3.3 per 10,0 from 2010 to 2019. During the same period, the density of dentists was 1.1 per 10,000 population.^1^ In the global context, the average density of medical doctors in the European Region is one doctor per 232 people, while in the Region of the Americas, there is one nursing and midwifery personnel per 121 people.^1^ In Nepal, annually more than 20 institutes train medical doctors and roughly 2000 to 2500 doctors are being produced.^2^ Yet, the density of medical doctors in Nepal is 8.67 per 10,000 population in 2021, which reflects both the migration of trained medical professionals to other nations and the need for doctors within the country.^3^

It aims to analyze the migration trends of healthcare professionals from Nepal, identify the underlying factors driving these trends, and assess the implications for Nepal's healthcare system and policy development.

## TREND ANALYSIS OF MEDICAL DOCTORS IN NEPAL

**Figure 1:**
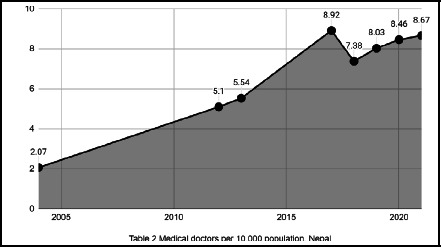
Density of Medical Doctors per 10,000 Population in Nepal (2004-2021).^4^

From 2004 to 2012, the density of medical doctors per 10,000 population in Nepal saw a significant rise, increasing from 2.07 to 5.1. This upward trend continued from 2012 to 2017, reaching a peak of 8.92. However, between 2017 and 2021, the trend exhibited fluctuations, with the number remaining at its highest in 2017 and experiencing a slight decline to 8.67 by 2021. The line chart depicting these trends highlights an upward trajectory in the density of medical doctors over the years, with notable growth up to 2017 followed by a period of variability (Figure 1).

Nepal Medical Council (NMC) receives four daily verification requests for foreign jobs.^5^ According to the Human Resources for Health (HRH) Projection in Nepal for 2079-2087 (2023-2031 AD), there is a deficit of 5,779 specialists, with an annual production growth rate of only 6.41% which highlights the widening gap in the doctor-to-population ratio.^6^ Currently, there is one doctor for every 850 people in the Kathmandu Valley, which surpasses the WHO's recommendation of 1 per 1000.^7^ However, in remote districts, this ratio drops significantly to 1 per 150,000.^7^

## TREND ANALYSIS FOR GOOD STANDING CERTIFICATES

A study done among the graduates of the oldest medical college, 73% pursued higher studies in the United States.^8^ From 2020 to 2023, the number of medical graduates pursuing careers abroad increased nearly 1.5 times from 869 to 2,318, indicating a rising demand for Good Standing Certificates (GSCs).^9^ Additionally, the Medical Education Commission issued 1,025 GSCs between April and December 2023.^6^ Data from 2020 to 2023 indicates an increasing trend of Nepali medical graduates obtaining good standing certificates (GSC) at NMC to pursue careers abroad rather than in Nepal (Table 1).

**Table 1:** Number of students pursuing a good standing certificate at NMC.^9^

SN	Country	2020	2021	2022	2023
1	USA	276 (25.39)	273 (18.18)	496 (22.66)	843 (32.65)
2	UK	162 (14.90)	319 (21.24)	592 (27.04)	505 (19.56)
3	Australia	62 (5.70)	66 (4.39)	109 (4.98)	166 (6.43)
4	India	62 (5.70)	159 (10.58)	106 (4.84)	186 (7.20)
5	Maldives	216 (19.87)	403 (26.83)	453 (20.69)	395 (15.30)
6	Other foreign country	91 (8.37)	107 (7.12)	198 (9.04)	223 (8.64)
	Total	1087	1502	2189	2582

This data depicts the number of doctors pursuing a Good Standing Certificate at the NMC from 2020 to 2023, categorized by country. The data shows trends in GSC applications, highlighting a significant increase in the number of stud ents from Nepal applying to the USA and the UK over the years.

## COMPARISON OF LICENSE EXAMINATION NUMBER IN UNDERGRADUATE AND POSTGRADUATE NUMBER

The comparison of the number of candidates appearing for undergraduate and postgraduate licensing examinations from 2018 to 2022 reveals a significantly lower number of candidates for the postgraduate level (Table 2).^9^ Approximately, the number of postgraduate candidates is roughly one-fourth of those taking the undergraduate licensing exams each year. For instance, in 2022, 5,687 candidates appeared for the undergraduate exam, while only 1,673 took the postgraduate exam. This stark disparity highlights the limited availability of postgraduate seats in Nepal, indicating fewer opportunities for undergraduate graduates to pursue further studies within the country. This lack of postgraduate opportunities has been a contributing factor to the increasing trend of students seeking postgraduate education abroad.

**Table 2:** License examination outcome for undergraduate and postgraduate levels (2018-2022).^9^

Year	**Bachelor Level License**			**Postgraduate Level Special License**		
	Appeared	Passed	Pass Rate	Appeared	Passed	Pass Rate
2018	4342	2408	55.5%	1182	745	63%
2019	5207	2672	51.3%	1374	872	63.5%
2020	2937	1813	61.7%	1163	755	64.9%
2021	2541	1354	53.28%	808	494	61.1%
2022	5687	2386	42%	1673	1234	73.8%

## FACTORS INFLUENCING MIGRATION OF HEALTHCARE PROFESSIONALS ABROAD

**Violence against medical professionals:** An article published in The Lancet found the prevalence of workplace violence to be 62-9% among Nepal Medical Council-certified physicians working in Nepal — a substantially high rate compared with data worldwide.^10^ Verbal, and physical violence has created an unsafe work environment, prompting medical professionals to seek opportunities in regions with better security measures. Continuous exposure to threats and assaults can lead to burnout, stress, and mental health issues, prompting migration for a safer and healthier work environment. The government made changes to the Health Workers and Health Organisations Act 2010 to reduce attacks on healthcare professionals and facilities. Despite the ordinance against violence against health workers of Nepal has been passed there is a need for stricter enforcement and accessible malpractice complaints.^11^

**Professional burnout:** Approximately 48% of healthcare professionals work over eight hours daily.^12^ Doctors often experience burnout from overwhelming workloads. This situation, combined with ill-equipped and understaffed hospitals and delayed referrals for serious cases, contributes to rising conflicts between doctors and patients.

**Low salaries and financial incentives:** Relatively lower salaries and limited financial incentives in Nepal, compared to other countries, is one of the significant factors pushing healthcare professionals to seek opportunities abroad. The AAMC (Association of American Medical Colleges) notes that the average Year one year salary of a medical resident is $ 63,400 in the year 2020, with an average yearly salary increase of 3%.^13^ Compared to this the salary of doctors in Nepal is Rs 5,84,844 per annum ($4357.32).^14^

**Job dissatisfaction:** A study by Gyawali et al. reported that 83.42% of the doctors in Nepal expressed dissatisfaction with their jobs with the least satisfaction in job privileges, career development, and human resources issues.^5^ The increasing rate of dissatisfaction is a matter of concern due to the direct impact of doctors' job satisfaction on patient safety and healthcare quality.

**Investment in the health sector:** The health sector was projected to grow by 6.5% in 2023/2024 but the sector's budget accounted for 5.8% in the same fiscal year.^15^ This discrepancy can result in insufficient resources, strained services, and challenges in maintaining quality care amidst growing demands.

**Political instability:** The influence of politics on the health system causes an unhealthy working environment for health professionals.

## IMPLICATIONS

**Improving Working Conditions and Reducing Violence**

To enhance working conditions and reduce violence against healthcare professionals, it is essential to properly implement existing ordinances designed for the safety of medical doctors in the workplace. This includes enforcing strict punishments, such as jail without bail, for perpetrators of violence. Additionally, addressing burnout involves increasing facilities and support services for doctors and their families, ensuring a safer and more supportive working environment.

**Expanding Postgraduate Seats and Employment Opportunities**

To address the growing demand for healthcare professionals, it is crucial to increase the proportion of postgraduate seats relative to undergraduate seats by the Medical Education Commission. Additionally, expanding the number of government medical doctor positions available through Loksewa can significantly enhance employment opportunities for graduates, contributing to a more robust and capable healthcare workforce.

**Addressing Salary Issues and Burnout**

It is essential to regularly review and adjust healthcare professionals' salaries to ensure they are competitive and reflective of their responsibilities. Establish systems to monitor and manage workloads, preventing excessive hours and reducing burnout.

**Political Stability**

Political stability is crucial for the effective implementation of healthcare policies and for securing adequate funding. Political instability can disrupt healthcare planning and result in inconsistent resource allocation. Stable governance supports continuous investment in the healthcare sector, enabling better planning and addressing systemic issues effectively.

## WAY FORWARD

Hence, healthcare workers are leaving Nepal for higher incomes, better living conditions, improved training, and safer work environments abroad, weakening the already fragile healthcare sector. Addressing these issues with targeted policies for workforce retention, improved working conditions, and better career development is crucial to sustaining Nepal's healthcare system and curbing this migration trend.

## DECLARATION OF INTERESTS

We declare no competing interests. The authors alone are responsible for the views expressed in this Viewpoint and they do not necessarily represent the views, decisions, or policies of the institutions with which they are affiliated.
